# The Role of Mineral Sea Water Bonding Process with Graphite-Aluminum Electrodes as Electric Generator

**DOI:** 10.1155/2019/7028316

**Published:** 2019-03-26

**Authors:** Satryo B. Utomo, Winarto, Agung S. Widodo, I. N. G. Wardana

**Affiliations:** ^1^Department of Electrical Engineering, Faculty of Engineering, Jember University, Indonesia; ^2^Department of Mechanical Engineering, Faculty of Engineering, Brawijaya University, Indonesia

## Abstract

The development of alternative ecofriendly electric energy production technology is very essential. The development of electric generator by using sea water element to boost electron jump in electrode has been studied. The aim of this research is to develop an electrode material composite that can collaborate with sea water to produce electricity. The electrodes tested are copper, aluminum, activated carbon, wood powder, and graphite electrode powder. The result shows that each electrode produces different voltage when interacting with sea water. It is caused by the patterns of the electron movement disrupted by the electron of sea water in producing a new compound. The composite of graphite-aluminum generates high electricity of about 580 mV compared to other materials. The electrical conductivity of graphite material depends on the particle size which is larger at smaller particle size. This research elucidates the role of sea water elements in boosting kinetic energy of delocalized electron on graphite resulting in an electron jump.

## 1. Introduction

Most of energy sources in the world are coming from fossil having harmful impact on the environment. It is very important to study alternative and ecofriendly energy sources. In the present study we develop the electric generator from the interaction of sea water with electrode developed from graphite, aluminum, copper, and wood particle. Many previous studies have used graphene electrodes to harvest electric energy from rain water droplet colliding on the electrode. The voltage produced by the impulse of rain water droplets on the electrodes is around 129 microvolts [1, 2]. Other researchers measure the response time of charge voltage during the interaction of the water droplet with the electrode. They found that the voltage generated is proportional to the water droplet kinetic energy [3]. Limitation may arise from rain power generator; i.e., the space for system installation is proportional to the amount of generated power. Therefore, electric power that can be generated by immersing electrode into sea water is developed in the present study. This type of power generation overcomes the space problems since it could be piled vertically. However, the electrode materials should be taken into account since they greatly affect the amount and condition of the charge transfer [4, 5]. Graphite material as an electrode has been widely applied in lithium batteries. The composition of graphite mixture can improve battery performance [6, 7]. This mechanism is associated with the electronic conductivity, the diffusion of electrode, and the ionic properties of electrode. Now, graphite is a phenomenal material. It is constructed by many layers of hexagonal carbon atoms structure with delocalized electron so that it is easy to bond with other elements. The graphite has been developed by various applications such as Pencil Graphite Electrodes (PGE) mixing with aniline and glycine [8], polymer materials [9], CuNS [10], enzyme [11], lithium batteries (LIBs) [12–14], solar cell [15–17], and sensor. Some researchers begin the development with modifying the size of and constructing combined nanomaterials for the electrode. There are many various nanocarbons having a smooth design for the development. Besides, they have upper electrochemical properties due to the improvement of the electrical conductivity and the stability of the mechanical strength.

The use of graphite electrode for electrical energy harvesting from sea water is promising. It has been reported in [1] that the rain water droplet can produce electric power of 295.48 pW/raindrop. However, the generation system should be installed horizontally, in right angel to the rain water drops which may occupy huge space. Therefore, new technology providing the same, or even higher, performance should be developed. In our study an electrode material using a combination of sea water and graphite is examined. The study is focused on the selection of new composite material that can produce and transfer electron when interacting with the sea water. The novelty of this study lies in disclosing the mechanism of sea water element to boost kinetic energy of delocalized electron on graphite resulting in an electron jump. Based on the description [1, 2], there are several steps that must be done. For the first step the present study discusses the characteristic of the oxidation reaction that occurs in sea water and in the atomic structure of graphite. The second step discusses the composite electrodes material that could accelerate the electron motion to generate higher electric energy.

The rest of this paper is structured as follows: Section 2 contained materials and methods to observe the voltage and current created from the interaction of sea water and the graphite composite electrode; Section 3 contained results and discussion, providing the mechanism of sea water chemical element to boost electron kinetic energy in graphite lattice to generate electron leap; Section 4 contained conclusions of the research finding.

## 2. Materials and Methods

### 2.1. Preparation of Material

**Figure 1:**
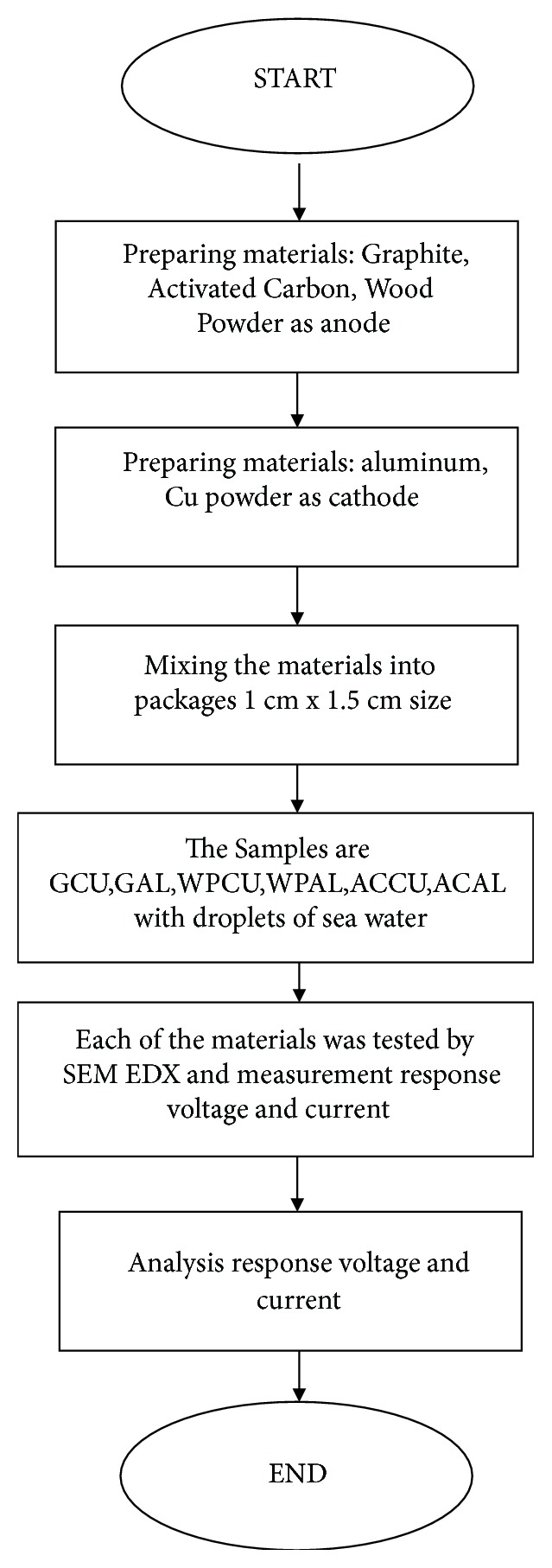
Flowchart of experiment.

The materials tested in this experiment were graphite [8], copper, aluminum foil, activated carbon, wood powder, and sea water (see Figure 1). The graphite was chosen for its special molecular structure related to the electron motion. The graphite was crushed with a mortar and was selected with 250 mesh. Then, it was packed in a plastic being ready to be synthesized. The copper on the PCB is crushed and filtered to form copper powder packaged in the plastic. The next stage is to prepare a transparent mica paper of 1.5 cm x 1 cm size plastered with double-sided tape to perfectly stick graphite powder on the mica. With this structure the sample was constructed into six compositions as

- graphite-copper (GCU),
- graphite-aluminum (GAL),
- wood powder-copper (WPCU),
- wood powder-aluminum (WPAL),
- activated carbon-copper (ACCU),
- activated carbon-aluminum (ACAL).

### 2.2. Chemical Element Sea Water

### 2.3. Characteristics of Material

The material was tested by SEM EDX, Hitachi, in order to measure the size of the graphite and the elements contained in the electrode. The voltage and the current generated by the material were tested by Sanwa Avometer.

### 2.4. Measurement of Material

**Figure 2:**
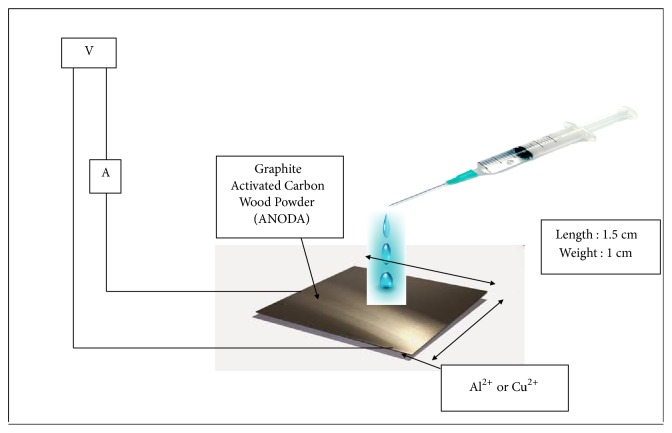
Method of response electricity measurement.

Figure 2 shows the measurement system. Measurement was done in many stages [18]. First, the conductivity of the material was measured using Sanwa DMM Avometer. The voltage was recorded by ACS712ELCTR-05B-T and microcontroller Arduino Uno. The Data Sheet ACS712ELCTR-05B-T has measurement range = ± 5 A and sensitivity = 185 mV/A. Current sensor has a read range signal from 0 (at 0V input) to 1023 (at 5V input) with a resolution of 0.0049V. The accuracy of current sensor is formulated as

(1) $$\begin{array}{c} I=\frac{\left(0.0049\times Vout-2.5\right)}{0.185}. \end{array}$$

The current and voltage measurements were obtained with a load of low power LED as shown in Figure 2. The uncertainty of the data was estimated from five measurement repetitions. The uncertainty of current and voltage data is ±5%.

Subsequently, measurement was done on the voltage generated by the electrode added with droplets sea water in the electrode. After that, each electrode was tested with SEM EDX, FEI S-50, and AMETEK.

## 3. Results and Discussion

To get better insight into the electric energy generation, the structure of graphite is presented using Avogadro software (see Figure 4). The graphite is constructed by many layers of hexagonal carbon atoms structure with delocalized electron so that it was easy to bond with other elements. The carbon atom with 0.76 atomic radius has electronegative value of 2.55 with ionization potential of 1086 kJ/mol and electron affinity of 122.3 kJ/mol [19].

Copper is an element which has one valence electron, 1.30 atomic radius, electronegative value of 1.9, ionization potential of 745 kJ/mol, and electron affinity of 118.3 kJ/mol, while aluminum is an element which has three valence electrons, 1.24 atomic radius with electronegative value of 1.61, ionization potential of 578 kJ/mol, and electron affinity of 42.6 kJ/mol [19].

Those parameters indicate that there is a difference in the distance between the atomic nucleus and the electron in carbon (C), copper (Cu), and aluminum (Al). The aluminum consists of three valence shells so it has stronger attractive electromagnetic force than the copper elements. It is because copper is composed of four shells and it had one free electron. Therefore, ionization potential energy of copper is greater than aluminum. Consequently, when they get energy, the electrons in aluminum are easily separated.

### 3.1. Conductivity of Materials

Table 2 is the material characteristics of graphite and copper. Graphite and copper were given barriers aids of 100 ohms and 1000 ohms with a voltage power supply of 10 volts. The current measured on copper was 99.6 mA and 9.86 mA. Besides, it has a constant response and it is influenced by the electrons on the copper electrons that are easily separated. The value measured in Table 2 proves that graphite and copper have electrical resistance. The smaller the electrical resistance in a material, the higher the speed of the flow of electrons. By using Ohm's law, it is shown that a copper conductor material has an average of 1.1 ohm barriers. The graphite material which has a median barrier of 2 megaohm is semiconductor material. On the other hand, activated carbon and wood powder have the lowest conductivity (0 – 0.1 mA) indicating that they are isolators.

**Figure 3:**
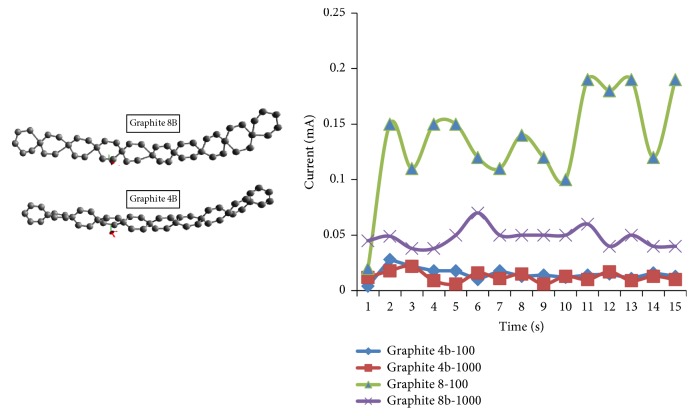
Graphite conductivity with a load of 100 and 1000 ohms.

Figure 3 shows graphite with different sizes. In SEM EDX test, graphite 4B contains 72.84 wt% carbon, 16.40 wt% oxigen, 3.80 wt% aluminum, and 5.18wt% silicon, while graphite 8B contains 73.90 wt% carbon, 15.90 wt% oxigen, 3.30 wt% aluminum, and 6.87 wt% silicon. Silicon in graphite 8B is greater than that in graphite 4B causing graphite 8B to easily release electrons. The graphite 8B and 4B were measured with 100-ohm; they had conductivity level between 0.1 mA and 0.2 mA for graphite 8B which is higher than that of graphite 4B, between 0.01 mA and 0.02 mA. In other words, the response of the current fluctuates (unstable). It is because the graphite is semiconductor so that the electron in dense graphite had a gap and a strong attractive force. However, the electrons in graphite are fragile and separable when they get energy from outside.

### 3.2. Mechanism of Graphite as Electric Generator

#### 3.2.1. Graphite without Sea Water

**Figure 4:**
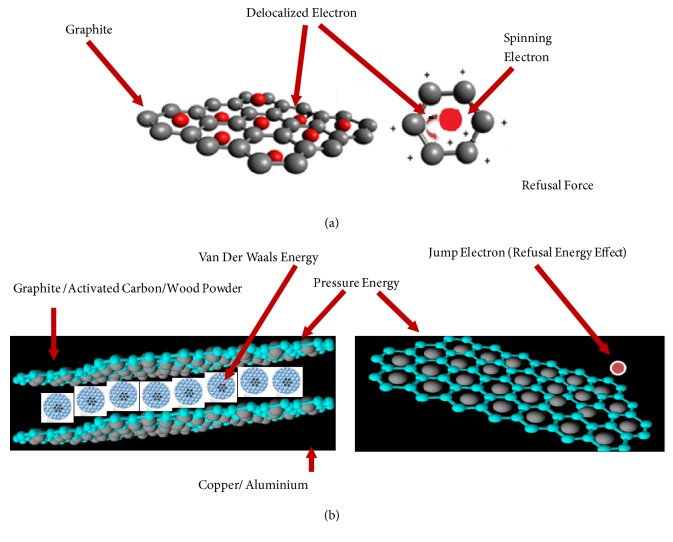
(a) Delocalized electron of graphite. (b) Graphite under pressure energy.

Graphite is tetrahedral structure of carbon atoms which consists of integrated benzene ring (see Figure 4). The electrons in the rings are delocalized due to the repulsive force among the electrons.  Figure 4(a) shows that when the electrons in the graphite elements are delocalized in a certain resonance speed, it could produce magnetic field. The magnitude of the magnetic field is affected by the distance of each electron and the spinning speed.

Each carbon in the graphite only bonds with three of them, the other carbons are set up in layers, and the fourth bond of each carbon is used to connect these layers. This bond is very long and the electrons are free moving along the structure of the areas. However, this electron's movement is very limited in one layer area so that the graphite could conduct the electricity. Besides, the graphite could collaborate with other elements, namely, copper and aluminum. Copper or aluminum has a positive ion and a high level of conductivity. When graphite with copper or with aluminum is placed in an electrode and it is given pressure of a certain frequency (Figure 4(b)), then there is a repulsive force among molecules since the graphite, copper, or aluminum has positive ions. The repulsive force could produce electricity.

Table 3 shows that the GCU has a small voltage (1mV-5mV) compared to GAL (10mV-25mV) when loaded with mass to create pressure on the layer of the material constantly in the time range of 4 to 6 seconds. Output voltage depends on the pressure or mass load. The GAL voltage is very dominant, 25 mV, and very sensitive when it is compared to the GCU, 2.5 mV. It is caused by the structure of the atoms; that is, the copper elements have four shells and one electron at the outer shell, and the energy required to remove the electron is the smallest. On the other hand, aluminum has three shells and three electrons at the outer shell. In order to release the outer shell of the electrons, it needs more energy than copper. Therefore, the positive graphite and the positive aluminum are in a symmetrical way and close to each other. It causes higher repulsive energy that creates induction between two materials (Figure 4(b)). In the case of graphite and copper atoms a particular compound could not be formed since the repulsive energy is lower. Table 3 shows that activated carbon and wood powder made from coconut shells have the lowest voltage (0-0.1 mV) indicating that they are isolators.

#### 3.2.2. Graphite with Sea Water

**Figure 5:**
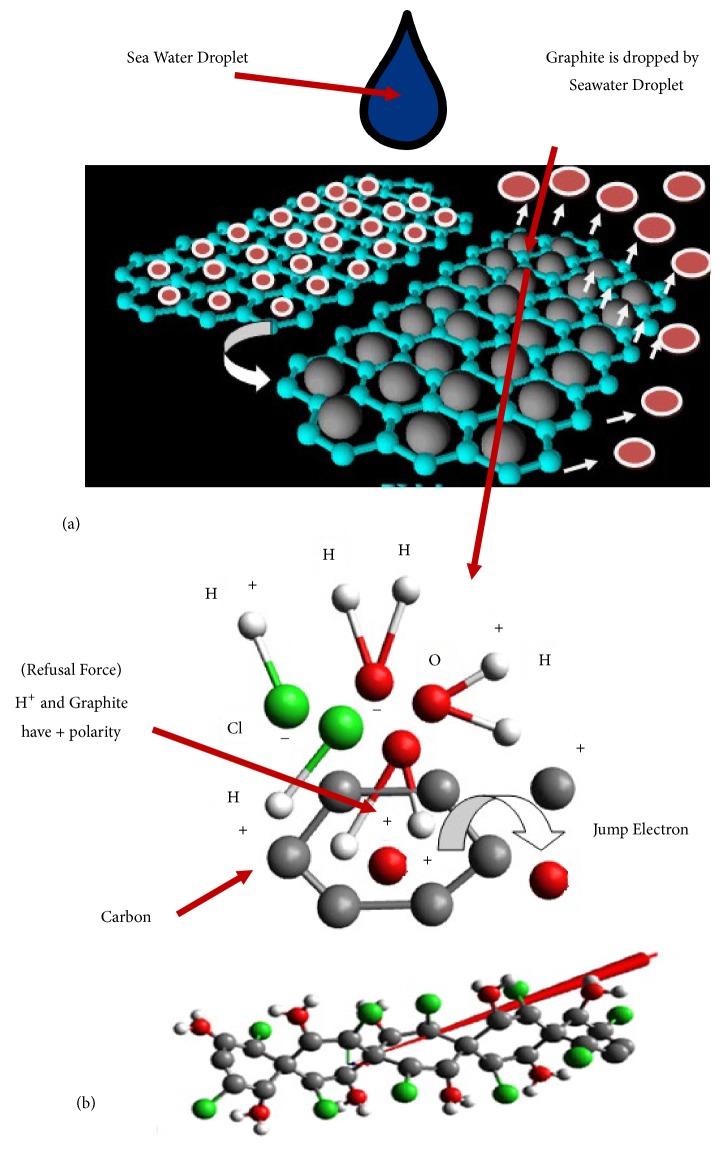
(a) The graphite is dropped by sea water. (b) The graphite and sea water element bonding.

When sea water interacts with graphite, the mobile electrons in graphite would be boosted by the potential energy of sea water elements. The energetic electrons then jump as shown in Figure 5(a). In Table 1, it is shown that sea water has the most dominant element of SO_4_^2−^ of 1000 Mg/L followed by 322.1 Mg/L of K and 57.45 Mg/L of Ca. The very few elements are sodium, Na of 14.32 Mg/L, followed by Cl of 8.88 Mg/L and Mg (magnesium) of 0.65 Mg/L. According to semiconductor theory, the electrons separated from the bond will be away from the hole that has a positive charge and will become free electrons that have a negative charge.

When sea water is in contact with the electrode, GCU or GAL, it causes the nonpolar and polar covalent bond as shown in Figure 4(b). The covalent nonpolar bond is formed when atoms of the sea water involved in bonding have the same electronegativity with graphite atoms, while for the polar bond the electronegativity is different. For the covalent polar bond, the positive graphite would attract the pair of Cl and OH since they have negative charge, while the other positive atoms, Mg, Ca, Cu, and Na, would form a new bond through a redox reaction.

As seen in Figure 5(b), the double bonds in the graphite generate delocalization of electron in order to have a stable energy level. When sea water interacts with graphite, the H_2_O would interrupt the free electrons in graphite that is in CC and CO. The stability of CO would be interrupted stronger because O atom would donate electrons to the C atom so that the electronegativity and the affinity among atoms are stronger and it could kick the electrons off. In addition, the leaped electrons are captured by Mg in sea water. Subsequently, elements of graphite and Cl build a covalent bond that has a different polarity. This improves the stability and the number of leaped electrons. The increased number of leaped electrons increases the power supply voltage.

#### 3.2.3. Role of Element of Sea Water in Producing Electric Energy

**Figure 6:**
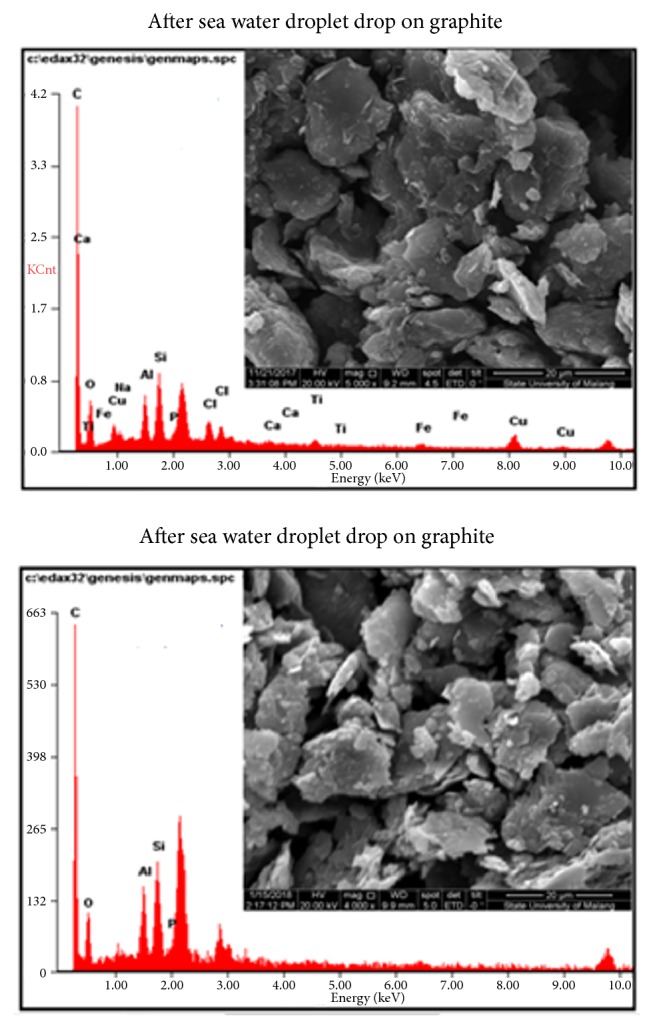
SEM EDX of graphite.

Figure 6 shows the result of SEM EDX test. The images showed that Mg and Ca are the dominant elements that could bond with graphite, while Na and Cl have the standard value. This indicates that there is a redox reaction. SEM EDX test confirms the process of hatching sea water into the electrode. The reaction of OH with Cl in the sea water is shown in the following reaction on a molecular basis according to [19] for Mg as

(2) MgOH2s+2HClaq→MgCl2aq+2H2Ol

The total ion reaction of (2) is

(3) MgOH2s+2H+aq+2Cl−aq→Mg2++2Cl−aq+2H2Ol

Since the negative Cl ion is attracted by positive graphite as explained in Figure 5(b) and confirmed by SEM EDX in Figure 6, then it became

(4) $$\begin{array}{c} Mg{\left(\;OH\;\right)}_{2}\left(\;s\;\right)+2H^{+}\left(\;aq\;\right)\to {Mg}^{2+}+2H_{2}O\left(\;l\;\right) \end{array}$$

The reaction of molecules for Ca is

(5) $$\begin{array}{c} Ca{\left(\;OH\;\right)}_{2}\left(\;s\;\right)+2HCl\left(\;aq\;\right)\to {CaCl}_{2}\left(\;aq\;\right)+2H_{2}O\left(\;l\;\right) \end{array}$$

The total ion reaction of (5) is

(6) CaOH2s+2H+aq+2Cl−aq→Ca2++2Cl−aq+2H2Ol

In this case, the negative Cl ion is also attracted by positive graphite (see Figures 5 and 6) so that it became

(7) $$\begin{array}{c} Ca{\left(\;OH\;\right)}_{2}\left(\;s\;\right)+2H^{+}\left(\;aq\;\right)\to {Ca}^{2+}+2H_{2}O\left(\;l\;\right) \end{array}$$

In the equations, it is explained that the element of positive charge in sea water binds with the element of negative charge of sea water when interacting with graphite. The charge of electrons on the left and right sides must be balanced, because they form new compounds.

**Figure 7:**
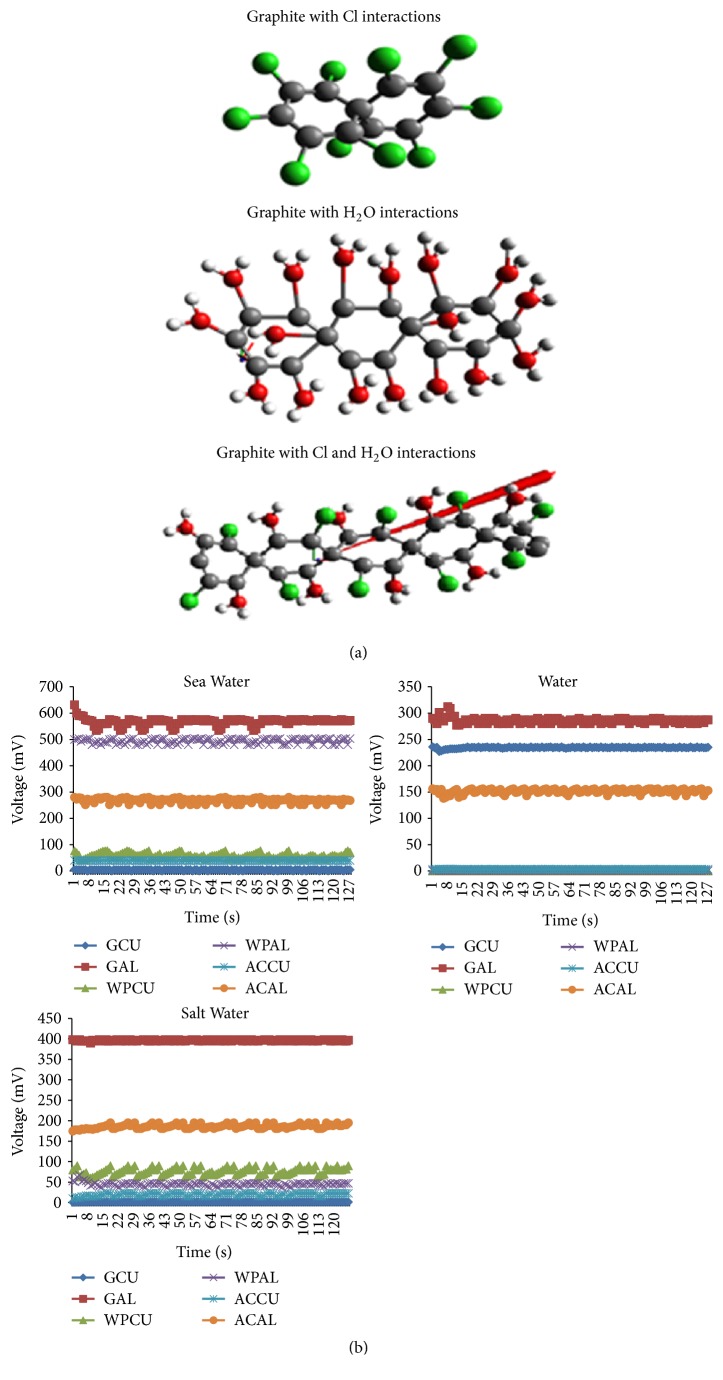
(a) Model of graphite interactions. (b) Response of electricity of sea water, water (H_2_O), and salt water.

**Figure 8:**
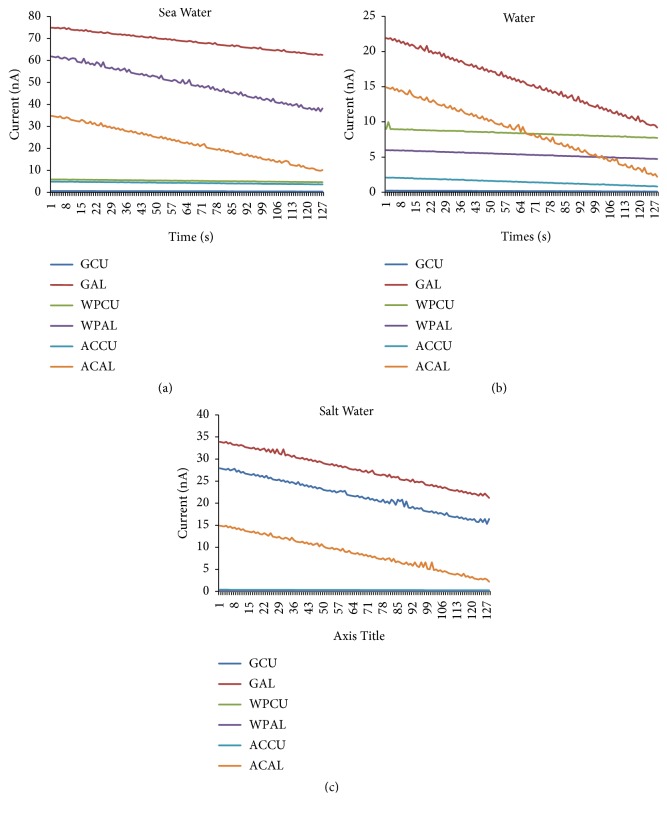
(a) Response of current of sea water. (b) Response of current of water (H_2_O). (c) Response of current of salt water.

Figures 7(b) and 7(a) show a voltage response of various materials when they are in contact with sea water. It is shown that the GAL, WPAL, and ACAL are able to produce a voltage of 580 mV, 490 mV, and 270 mV, and Figure 8(a) shows respective current of 75 nA, 62 nA, and 35 nA. On the other hand GCU, WPCU, and ACCU are only able to produce a voltage of 5 mV, 55 mV, and 39 mV, and Figure 8(a) shows respective current of 0.63 nA, 5.9 nA, and 4.2 x nA. This shows that with sea water the Al composite electrode improves voltage almost 10-fold as high as that of Cu because aluminum reacts with the compound contained in the sea water, hydrochloric acid (HCl), and also with OH^−^ compound to become hydroxide Al (OH)_3_. The positive aluminum ion would attract the negative chloride ion (Cl). The movement of electrons which collaborate with each other caused the electricity so that the aluminum based composite electrode and sea water became good electric generator.

The Al reacts with hydroxide when being in contact with sea water containing Cl as the following equation shows.

(8) $$\begin{array}{c} Al{\left(\;OH\;\right)}_{3}\left(\;s\;\right)+3HCl\left(\;aq\;\right)\to {AlCl}_{3}\left(\;aq\;\right)+3H_{2}O\left(\;l\;\right) \end{array}$$

The total ion reaction of (8) is

(9) AlOH3s+3H+aq+3Cl−aq→Al3++3Cl−aq+3H2Ol

Again, the negative Cl ion is attracted by positive graphite as explained in Figure 5(b) and confirmed by SEM EDX in Figure 6. Then it became

(10) $$\begin{array}{c} Al{\left(\;OH\;\right)}_{3}\left(\;s\;\right)+3H^{+}\left(\;aq\;\right)\to {Al}^{3+}+3H_{2}O\left(\;l\;\right) \end{array}$$

Equations (8), (9), and (10) indicate that when the GAL electrode is in contact with sea water, the aluminum can react with elements in sea water. H_2_O elements break down when interacting with graphite and bind with OH-elements, so that Al reacts with Cl releasing H. This reaction causes graphite/aluminum to produce electron jumps. Copper on the other hand does not react with Cl elements in sea water, because its level of reactivity is different. Graphite has voltage higher than wood powder and activated carbon. It depends on the amount of Cl that it reacts with. SEM EDX test shows that graphite contains Cl higher (2.08 wt%) than activated carbon (1.07 wt%) and wood powder (0.66wt%).

As shown in Figure 8(b) as the electrodes are infused with* water* (H_2_O), the GAL, WPAL, and ACAL are able to produce a current of 34 nA, 0.076 nA, and 15 nA, but GCU, WPCU, and ACCU are only able to produce a current of 28 nA, 0.013 nA, and 0.37nA. Figure 8(b) shows that GAL and ACAL produce a voltage of 300 mV and 138 mV, respectively, which is half of those at sea water. But the voltage produced by GCU increases drastically from 5 mV in sea water to 230 mV in water. On the other hand WPAL, WPCU, and ACCU produce a voltage of 0.1 mV, 0.8 mV, and 3.4 mV, respectively. It is because aluminum and carbon compounds react with H^+^ (see Figure 5(b)).

As the electrodes are infused with salt water as shown in Figure 7(b), the GAL and ACAL produce voltage higher than those at water (Figure 7(b)), i.e., 400 mV and 180 mV, respectively. WPAL, WPCU, and ACCU produce a small voltage of 30 mV, 60 mV, and 20 mV, respectively, and GCU produces almost 0 mV. It is because of aluminum compounds that react with Cl as explained in Figure 5(b).  Figure 8(c) shows the GAL, WPAL, and ACAL produce a current of 22 nA,6 nA, and 15 nA, but GCU, WPCU, and ACCU are only able to produce current of 0.23 nA, 9 nA, and 0.21 nA.

**Figure 9:**
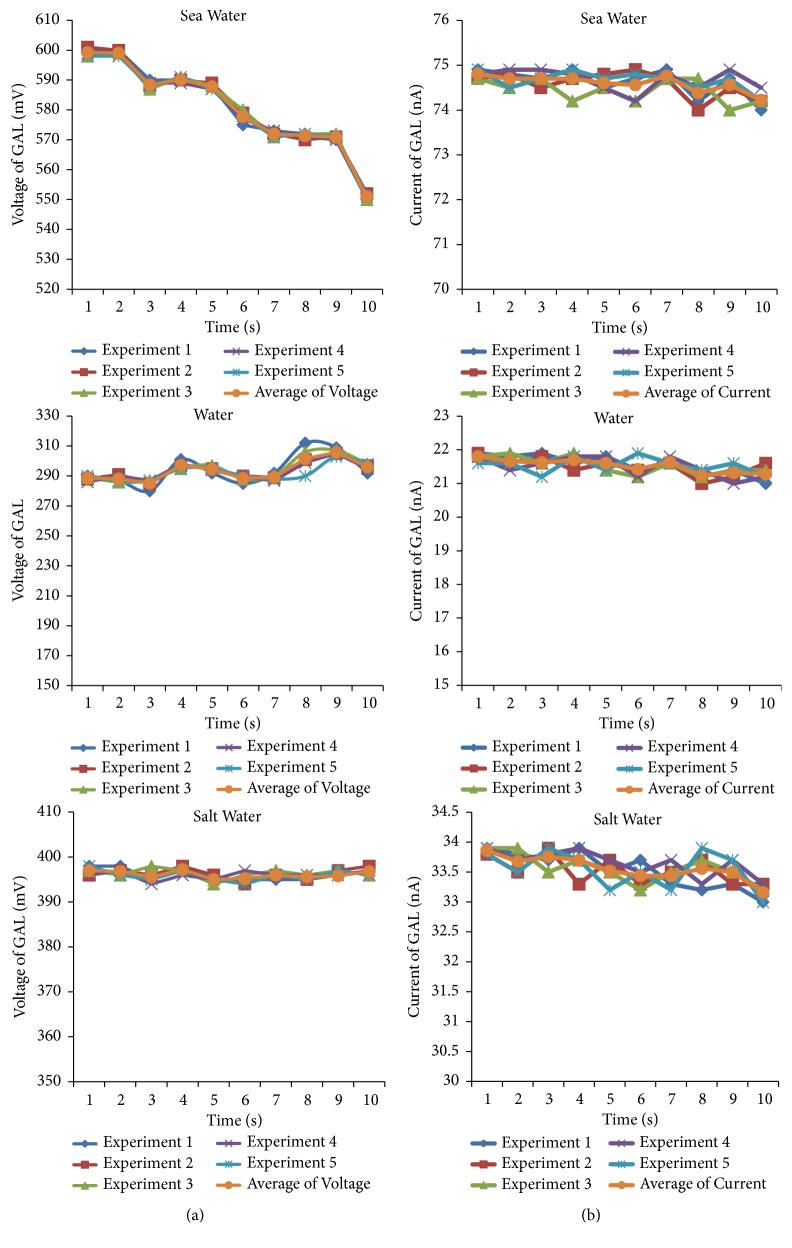
(a) Range of GAL voltage. (b) Range of GAL current.

Figure 9(a) shows a voltage response for five measurement repetitions. The voltage of GAL when it is in contact with sea water at 10 seconds is in the range of 551mV–558 mV. It is caused by the fact that graphite is able to store energy when the electrode interacts with H, OH, and Cl elements. However, when materials are in contact with water (H_2_O), droplet GAL voltage is in the range of 289 mV-305 mV. It is becuase the energy stored by graphite is only coming from the interaction of electrode with H and OH. In the other case, when materials are in contact with salt water, droplet GAL voltage is in the range of 394mV -398mV. It is due to energy stored by the graphite coming from its interaction with Cl only.

Figure 9(b) shows a current response for five measurement repetitions. It is shown that GAL current is in the range of 74.2 nA–74.9 nA. However, when materials are in contact with water (H_2_O) the GAL current is 21nA-21.9 nA. In the case of materials being in contact with salt water, the GAL current is 33nA- 33.9 nA. Figures 9(a) and 9(b) show that the voltage and current data are fairly consistent within ± 5% uncertainty.

In our study, it can be concluded that Cl and H_2_O elements have important role in generating electricity using composite electrodes from graphite, activated carbon, Al, Cu, and wood particle infused with sea water. The composite GAL electrode produces the highest electrical energy in sea water since it reacts with Cl and H_2_O. The other reason is that ionization electron affinity of aluminum is lower than copper. So, when they get energy the electrons in aluminum are easily separated. SEM EDX test in Figure 6 shows that graphite can bind with Cl element most widely. The reaction of elements in sea water with electrode material is confirmed by the transient current during the contact between sea water and the electrode sample. The reduction of the current with time in Figure 7 indicates the reduction of the number of Cl reactants to become new bonding products.

When sea water, H_2_O, and salt water are in contact with graphite, it loses electrons (electron jump). Figures 9(a) and 9(b) show voltage and current responses of graphite for five measurement repetitions at 10 seconds. This suggests that the elements in sea water give energy for graphite. So, graphite can form new compound by binding with H, OH^−^, and Cl^−^. In rain water droplets, it is possible to harvest energy from rain water utilizing graphene electrode [1, 2]. However, the energy is harvested with piezoelectric system positioned horizontally. Consequently, very huge space is needed. In our study, new different techniques are developed to boost electron energy, which make electron leap out of graphite/aluminum electrode utilizing chemical elements in sea water, especially H, OH^−^, and Cl^−^. Our result shows that any single sea water droplet could produce electric potential of around 580 mV and energy of about 43000p Watt/droplet. The power generated can be increased even much more by increasing the electrode surface area and immersing it in sea water. Moreover, the occupation of the horizontal space area in rain water electric energy harvesting could be overcome since the electrode in the present study could be piled vertically in sea water.

## 4. Conclusions

This study aims to find the type of material that can generate electrical energy when dropping sea water and study the behavior of electrons in forming new bonds. The process of electrical energy in graphite material is influenced by several factors, namely:

- The H_2_O element has a role in interrupting the delocalized electron process in graphite resulting in an electron jump. The impact of the jump can generate electrical energy.
- When graphite interacts with sea water, the graphite would have positive charge, so it can bind with the Cl element. This is due to the Vander Waals force and redox reactions. This effect causes graphite to have capacitor characteristic.
- The combination of GAL generates voltage of about 580 mV, current of 75 nA, and energy of about 43000 p Watt/droplet compared to other materials since the electron bonding energy in the aluminum is very small. So the electron is easily disturbed by the molecule of sea water (H_2_O, Cl) to move easily.
- Sea water is able to change characteristic of piezoelectric material into electric generator. This is due to the movement of electrons in redox reaction.

## Figures and Tables

**Table 1:** Chemical Element in sea water from Jember, East Java, Indonesia.

No.	Element	Analysis Results	Analysis Methods	
		(Mg / L)	Reagent	Method
1	Ca	57.45 + - 0:04	HNO_3_	AAS
2	K	322.1 + -0.03	HNO_3_	AAS
3	SO_4_^2−^	1000+ -0.00	HCl- BaCl_2_	spectrometry
4	Cl	8.88 + -0.00	AgNO_3_	Argentometry
5	Mg	0.65 + -0.00	HNO_3_	AAS
6	Na	14.32 + -001	HNO_3_	AAS

**Table 2:** Current measurement of materials.

Load	Current (mA)				
	Graphite-4B	Activated Carbon	Graphite-8B	Wood Powder	Cu
100 ohm	0.018	0.0001	0.15	0	99.6
1000 ohm	0.009	0.0001	0.07	0	9.86

**Table 3:** Response voltage of materials.

Voltage (mV)						
Energy (Joule)	GCU	GAL	WPCU	WPAL	ACCU	ACAL
mass = 0.0075 kg h = 30 cm E= 0.002205 (J)	3.97	10.1	0.1	0.2	0.1	0.2
	3.1	10.2	0.1	0.2	0.1	0.2
	6	20.4	0.1	0.2	0.1	0.2
	2.59	20.2	0.1	0.2	0.1	0.2
	5.38	19.8	0.1	0.2	0.1	0.2
	2.7	24.23	0.1	0.2	0.1	0.2
	4.08	12.2	0.1	0.2	0.1	0.2
	2.1	15.08	0.1	0.2	0.1	0.2
	4.44	7.8	0.1	0.2	0.1	0.2
	3.43	7.9	0.1	0.2	0.1	0.2

mass = 0,1 kg h = 30 cm E = 0.00294 (J)	1.34	16.87	0.1	0.2	0.1	0.2
	1.29	17.59	0.1	0.2	0.1	0.2
	1.36	36.04	0.1	0.2	0.1	0.2
	0.19	14.2	0.1	0.2	0.1	0.2
	0.1	15.11	0.1	0.2	0.1	0.2
	0.14	9.94	0.1	0.2	0.1	0.2
	0.42	19.3	0.1	0.2	0.1	0.2
	0.95	30.39	0.1	0.2	0.1	0.2
	0.37	26.16	0.1	0.2	0.1	0.2
	0.15	21.68	0.1	0.2	0.1	0.2

## Data Availability

The data used to support the finding of this study are available from the corresponding author upon request.
